# Soil acidification drives a massive accumulation of nitrite as a mechanistic bottleneck to inhibit maize growth and productivity

**DOI:** 10.3389/fmicb.2026.1830432

**Published:** 2026-09-18

**Authors:** Hang Luo, Di Xu, Yifan Wang, Tianyi He, Lu Zhang, Dan Jiang, Yang E., Haijun Hu, Wenfu Chen

**Affiliations:** 1 National Biochar Institute of Shenyang Agricultural University, Shenyang, China; 2 Zunyi Normal College, Zunyi, China

**Keywords:** functional decoupling, Mollisol, nitrification bottleneck, nitrite accumulation, phytotoxicity, soil acidification

## Abstract

Soil acidification poses a critical threat to global agricultural sustainability, particularly in high-buffering Mollisol (black soil) ecosystems. However, the microbially mediated biochemical mechanisms underlying nitrogen (N) transformation blockages and subsequent phytotoxicity under acid stress remain poorly understood. Here, using a controlled soil acidification gradient (pH 2.0–7.0) combined with 16S rRNA gene sequencing, functional profiling (FAPROTAX), quantitative PCR (qPCR), and chemical N speciation, we unravel a key microbially driven mechanism governing acid-induced crop failure. Our findings demonstrate that soil acidification imposes strong deterministic environmental filtering (βNTI approaching +2) on the rhizosphere microbiome, driving a drastic collapse in microbial phylogenetic diversity (Chao1 index). This structural transition severely alters N-cycling functional guilds, inducing a profound functional bottleneck. In strong acid treatments (pH 2.0–4.0), ammonium (NH_4_^+^) and nitrate (NO_3_^–^) concentrations plummeted to 0.25-fold and 0.20-fold of those in circumneutral soils, respectively. Crucially, nitrite (NO_2_^–^-N) underwent a 5.85-fold hyper-accumulation, reaching up to 28.52 mg kg^–1^. At the molecular level, this accumulation was driven by a transcriptional and functional decoupling between nitrifying cohorts: while ammonia oxidation genes (*amoA/amoB* in *AOA* and *AOB*) were upregulated or sustained under acidity, nitrite oxidoreductase (*NxrC*) expression and gaseous denitrification pathways were almost completely suppressed. To decouple nitrite phytotoxicity from direct proton (H^+^) stress, exogenous sodium nitrite (NaNO_2_) was spiked into neutral soil (pH 7.0) matching the acidification gradient (1.75–28.52 mg kg^–1^; M7–M2). Dose-response validation revealed that NO_2_^–^-N exceeding 13.21 mg kg^–1^ exerted severe phytotoxicity, significantly inhibiting maize germination, leaf area, and height, with total plant mortality occurring at ≥27.78 mg kg^–1^ NO_2_^–^-N. Collectively, this study establishes that acid-induced functional decoupling of the nitrifying community causes a massive NO_2_^–^-N accumulation, acting as an relatively overlooked, independent phytotoxic driver of crop failure in acidified soils. These insights demonstrate that restoring acidified agroecosystems requires shifting from simple pH neutralization to integrated microbiome management aimed at relieving N-transformation bottlenecks.

## 1 Introduction

The relentless expansion of the global population, compounded by escalating environmental pollution and the progressive contraction of arable land (Xiao et al., 2026), has reinstated food security as a preeminent global challenge. To meet soaring food demands, agronomists worldwide have historically relied on the intensive application of nitrogenous (N) fertilizers (Zhang et al., 2015). However, the nitrification of ammonium-based fertilizers liberates significant quantities of protons (H^+^), serving as a primary anthropogenic driver of soil acidification (Tian and Niu, 2015). Chronic N over-loading not only precipitates a decline in topsoil pH but also triggers a deleterious shift in microbial community structures, ultimately threatening the stability of global food systems (Li et al., 2021; Munyaneza et al., 2024).

In Northeast China, the Mollisol (black soil) region constitutes one of the world’s most critical breadbaskets (Xu et al., 2024; Li et al., 2025). These soils are traditionally characterized by their exceptionally high soil organic matter (SOM) content, which provides a robust pH buffering capacity compared to other pedological types (Curtin and Trolove, 2013; Li et al., 2026). Under moderate N application, the vast humic substances and functional groups within the black soil matrix can sequester excess protons, thereby partially mitigating N-induced acidification (Xu et al., 2024). However, recent observations indicate that the buffering threshold of these Mollisols is being exceeded due to intensified farming. Persistent N over-fertilization has begun to strip the soil of its base cations and accelerate SOM mineralization, altering microbial metabolic feedbacks. This dealkalization process diminishes the soil’s resilience, leading to systemic acidification even in SOM-rich environments. Consequently, the depletion of the buffering capacity in black soils triggers secondary crises, such as aluminum (Al^3+^) toxicity and heavy metal mobilization, necessitating urgent investigations into the biochemical shifts occurring within this unique pedological context (Shetty et al., 2021).

The systematic evolution of soil microbial communities serves as a critical conduit for biologically mediated acidification (Philippot et al., 2024). Upon surplus N input, nitrification exhibits a transient surge, generating substantial protons and inducing short-term soil acidification in topsoil (Dong et al., 2022). As acidic stress persists, the coupling of carbon and nitrogen cycling is destabilized, often accompanied by a successional shift toward acid-tolerant fungi. Crucially, shifts in microbial metabolic trajectories facilitate a regime shift from gradual acidification to irreversible degradation. While the prevailing consensus attributes crop yield decline primarily to Al^3+^ phytotoxicity (Singh et al., 2017; Chauhan et al., 2021), the potential for acidification-induced biochemical toxins, specifically those arising from impaired N-metabolism, remains largely unexplored in high-SOM Mollisols.

Despite extensive documentation of soil degradation, the structural and functional response of rhizosphere microbial communities to acidification within a pedologically consistent Mollisol background remains a critical knowledge gap. To address this, we utilized high-quality Mollisols from Northeast China to construct a comprehensive acidification gradient, modulating soil pH from 2.0 to 7.0 via precise titration. By integrating high-throughput sequencing with rigorous chemical analyses, we systematically characterized the bifurcated impact of acidification on the soil-microbe-plant nexus. Furthermore, by correlating these shifts with the agronomic performance of maize (*Zea mays L.*), we aimed to decipher whether the suppression of N-transforming functional groups leads to the lethal accumulation of nitrite (NO_2_^–^) as an alternative, yet overlooked, phytotoxic pathway in acidified black soils.

## 2 Materials and methods

### 2.1 Construction of soil acidification gradient and plant cultivation

This study utilized typical Mollisols (Black soil) from Northeast China as the experimental substrate, with baseline physicochemical properties and elemental composition detailed in Supplementary Tables 1, 2. To establish a standardized acidification gradient, irrigation water was precisely titrated with acetic acid to achieve a pH range of 2.0–7.0. Each soil microcosm was subsequently saturated with 2,000 mL of the pH-adjusted solution, while the edaphic pH dynamics were monitored *in situ* using high-precision pH probes (SevenDirect mix, Mettler toledo, Switzerland) to ensure the final soil acidity stabilized within the target intervals for subsequent investigation. *In-situ* soil pH monitoring was conducted throughout the entire growth period of maize. The final soil pH values are listed in Supplementary Table 1. The maize cultivars (Xianyu 335), sourced from Shenyang Agricultural University, were employed as the biological model and sown into the pre-conditioned soils at a density of three seeds per hole. To ensure statistical robustness and mitigate experimental error, all pot trials were executed with five independent biological replicates for each pH treatment, providing a rigorous framework for assessing the impact of soil acidification on plant development and microbial ecology. Five biological replicates were performed for each treatment.

### 2.2 Agronomic trait assessment

The phenotypic characterization of maize morphological and yield-related traits was conducted through standardized agronomical assessments to ensure high-precision data acquisition. Plant height was precisely quantified using a telescopic measuring rod from the soil surface to the apex of the tassel, while stem robustness was evaluated by measuring the major and minor axes of the second and third basal internodes using digital Vernier calipers to account for stem ellipticity. The Leaf Area Index (LAI) is defined as the total one-sided green leaf area per unit of horizontal ground surface area:


$$\text{LAI}=\frac{\text{Leaf}\,\text{Area}\,\left({\text{m}}^{2}\right)}{\text{Ground}\,\text{Area}\,\left({\text{m}}^{2}\right)}$$

Leaf area = Length × Width × 0.75. For pot experiment in our experiment, the ground area is fixed at 0.13 m^2^. To evaluate grain productivity and sink strength, the hundred-kernel weight was determined by randomly sampling three independent cohorts of 100 well-developed kernels, with final masses standardized to a uniform 14% moisture content to ensure comparative accuracy across varying environmental treatments.

### 2.3 DNA extraction, PCR amplification and sequencing

Maize plants were cultivated in plastic pots measuring 40 cm in diameter and 35 cm in height. During the ripening stage, rhizosphere soil was collected by gently shaking the maize roots for high-resolution 16S rRNA gene profiling on the Illumina HiSeq 2500 platform. Genomic DNA was isolated from each sample using the FastDNA SPIN Kit and quantified via the PicoGreen dsDNA Assay Kit before being standardized to a concentration of 3.5 ng μL^–1^. The V5–V7 hypervariable regions of the bacterial 16S rRNA gene were targeted for amplification using the degenerate primer pair 799F and 1193R in a 30 μL reaction system containing 0.75 U PrimeSTAR HS DNA polymerase and 0.2 mM dNTPs. This primary amplification phase consisted of an initial denaturation at 98 °C for 30 s, followed by 25 cycles of stringent thermal conditions (98 °C for 10 s, 55 °C for 15 s, and 72 °C for 60 s), and a final 5-min extension at 72 °C. Upon verifying the absence of contamination in negative controls, triplicate PCR products were pooled and purified using the AMPure XP system, subsequently standardized to 10 ng μL^–1^ for a secondary nested PCR. This second step employed barcoded primers for an additional eight cycles under identical thermal profiles to incorporate sequencing adapters. The resulting amplicons were resolved on 1.2% agarose gels, recovered via the QIAquick Gel Extraction Kit, and equimolarly pooled at 200 ng per sample following precise PicoGreen quantification. The final consolidated libraries underwent double purification with Agencourt AMPure XP beads to ensure optimal sequence quality and cluster generation for the subsequent sequencing run.

To validate the FAPROTAX analysis results, the *AOA*, *AOB* and *NxrC* gene were analyzed by qPCR. DNA was isolated from the soil (0.5 g) using soil nucleic acid reagent (Takara, Dalian, China) following the manufacturer’s instructions. The DNA quality and concentration were tested by gel electrophoresis and a NanoDrop spectrophotometer (NanoDrop Technologies, Wilmington, DE, USA). The integrity and uniformity of the DNA in each sample were confirmed by the amplification of Arch-amoA-F/Arch-amoA-R (forward primer: GATGGAGACTAGAACTGGAC; reverse primer: CTTACCAGCACCAAGGATGA), amoB-1F/amoB-2R (forward primer: GGAACGGCATCGGACCGAAG; reverse primer: AGCTCGATTCACGCTAGACGA) and NxrC1-F/NxrC1-R (forward primer: CACTCTTGCTGGACGTCA, reverse primer: CATATCCACAACCACGTG). Real-time quantitative PCR (qPCR) was performed under the following thermocycling conditions: an initial denaturation step at 95 °C for 10 min, followed by 40 °C of denaturation at 95 °C for 15 min and combined annealing/extension at 60 °C for 30 min, during which fluorescence signals were captured. To confirm target specificity and rule out non-specific amplification or primer-dimers, a melt curve analysis was conducted immediately following amplification by continuously monitoring fluorescence while ramping the temperature from 60 °C to 95 °C at 0.5 °C/s.

### 2.4 Bioinformatics analysis on 16S rRNA gene profiling

The 16S rRNA genesequences were processed using QIIME v.1.9.1, USEARCH v.10.0 and in-house scripts17. Metadata are given in Supplementary Table 3. The quality of the paired-end Illumina reads was checked by FastQC v.0.11.5 and processed in the following steps by USEARCH: joining of paired-end reads and relabeling of sequencing names (-fastq_mergepairs); removal of barcodes and primers (-fastx_truncate); filtering of low-quality reads (-fastq_filter); and finding non-redundancy reads (-fastx_uniques). Unique reads were clustered into OTUs with 97% similarity. The representative sequences were picked by UPARSE46. OTUs were aligned to the SILVA 132 database to remove sequences from chimera and host plastids47. The OTU table was generated by USEARCH (-otutab). The taxonomy of the representative sequences was classified with the RDP classifier48. Diversity analysis was carried out using self-Rscript and QIIME2. Analysis of the differential OTU abundance and taxa was performed using Wilcoxon rank sum tests based on OTUs with median relative abundance from each genotype >0.2%, and corresponding *P*-values were corrected for multiple tests using a FDR set at 0.05. Functional annotations of prokaryotic taxa were carried out using FAPROTAX v.1.1.

### 2.5 Quantification of soil nitrogen fractions

Soil nitrogen speciation, including ammonium (NH_4_^+^-N), nitrate (NO_3_^–^-N), and nitrite (NO_2_^–^-N), was quantitatively determined following extraction with a 2M KCl solution at a soil-to-solvent ratio of 1:5 (w/v), where samples were subjected to 1 h of orbital shaking at 200 rpm followed by immediate filtration through quantitative ashless filter paper. Filtrates were stored in darkness at 4 °C, and NO_2_^–^-N analysis was finished within 24 h post-extraction to mitigate chemical degradation of nitrite in acidic extracts. Spike-recovery pre-tests using representative experimental soils were performed by spiking NO_3_^–^-N standard solution before KCl extraction, achieving acceptable NO_3_^–^-N recoveries of 85%–115%. The concentration of NH_4_^+^-N in the resulting extracts was measured using the indophenol blue colorimetric method, involving the reaction of ammonium with sodium salicylate and sodium hypochlorite in the presence of sodium nitroprusside to form a blue-colored complex under alkaline conditions. Concurrently, NO_3_^–^-N was quantified via the ultraviolet spectrophotometric screening method at dual wavelengths of 220 nm and 275 nm to account for organic matter interference, or alternatively, via the vanadium chloride (VCl_3_) reduction method to ensure high-sensitivity conversion of nitrate to nitrite for colorimetric detection. Most crucially, the quantification of NO_2_^–^-N was executed utilizing the Griess-Ilosvay diazotization-coupling reaction, where nitrite reacts with sulfanilamide and *N*-(1-naphthyl)-ethylenediamine dihydrochloride under acidic conditions to form a stable pink azo dye measured at 543 nm. All spectrophotometric measurements were performed on a microplate reader or a dual-beam UV-Vis spectrophotometer, with final concentrations calibrated against high-purity analytical standards and standardized to the dry weight of the soil to ensure inter-sample comparability and analytical rigor.

### 2.6 Assessment of nitrite-induced phytotoxicity

To systematically decouple the specific phytotoxic effects of nitrite (NO_2_^–^-N) accumulation from the confounding influence of chemical proton stress (H^+^ toxicity), a controlled validation experiment was established using neutral soil (pH = 7.0). Exogenous sodium nitrite (NaNO_2_, analytical grade, China National Pharmaceutical Group Shanghai Co., China) was applied to the neutral soil. The dosage of NaNO_2_ was meticulously calibrated to match the exact soil NO_2_^–^-N concentrations observed across the acidification gradient (pH 2.0–7.0), simulating the nitrite accumulation environment of acidified soils within a circumneutral matrix. The treatments were designated as M7–M2, corresponding to target soil NO_2_^–^-N concentrations of 1.75, 6.52, 13.21, 17.99, 28.52, and 27.78 mg kg^–1^ (representing baseline/control through maximum acidification levels).

## 3 Results

### 3.1 Impact of soil acidification on maize growth and agronomic performance

To elucidate the impact of soil acidification on crop development, we systematically evaluated the agronomic traits of maize across a localized pH gradient (pH 2.0–7.0) in pot experiments. Extreme acidity (pH ≤ 4) triggered total seedling mortality, rendering phenotypic measurements unviable for these extreme treatments. Among the surviving cohorts (pH 5.0–7.0), both plant height and stem diameter exhibited a robust positive correlation with soil pH, culminating in peak values of 176.82 ± 13.41 cm and 2.64 ± 0.18 cm, respectively, at a neutral pH of 7 (Figures 1a, b). Regarding reproductive traits, ear diameter remained statistically invariant across pH 5.0–7.0 (Figure 1c), panicle length, particles per row, and 100-seed weight were significantly suppressed by increasing acidity (Figures 1d–f). Notably, the highest grain yield (43.46 g) and optimal agronomic architecture were consistently recorded at pH 7, identifying it as the threshold for maximizing maize fitness and highlighting the catastrophic consequences of acidification-induced physiological impairment.

**FIGURE 1:**
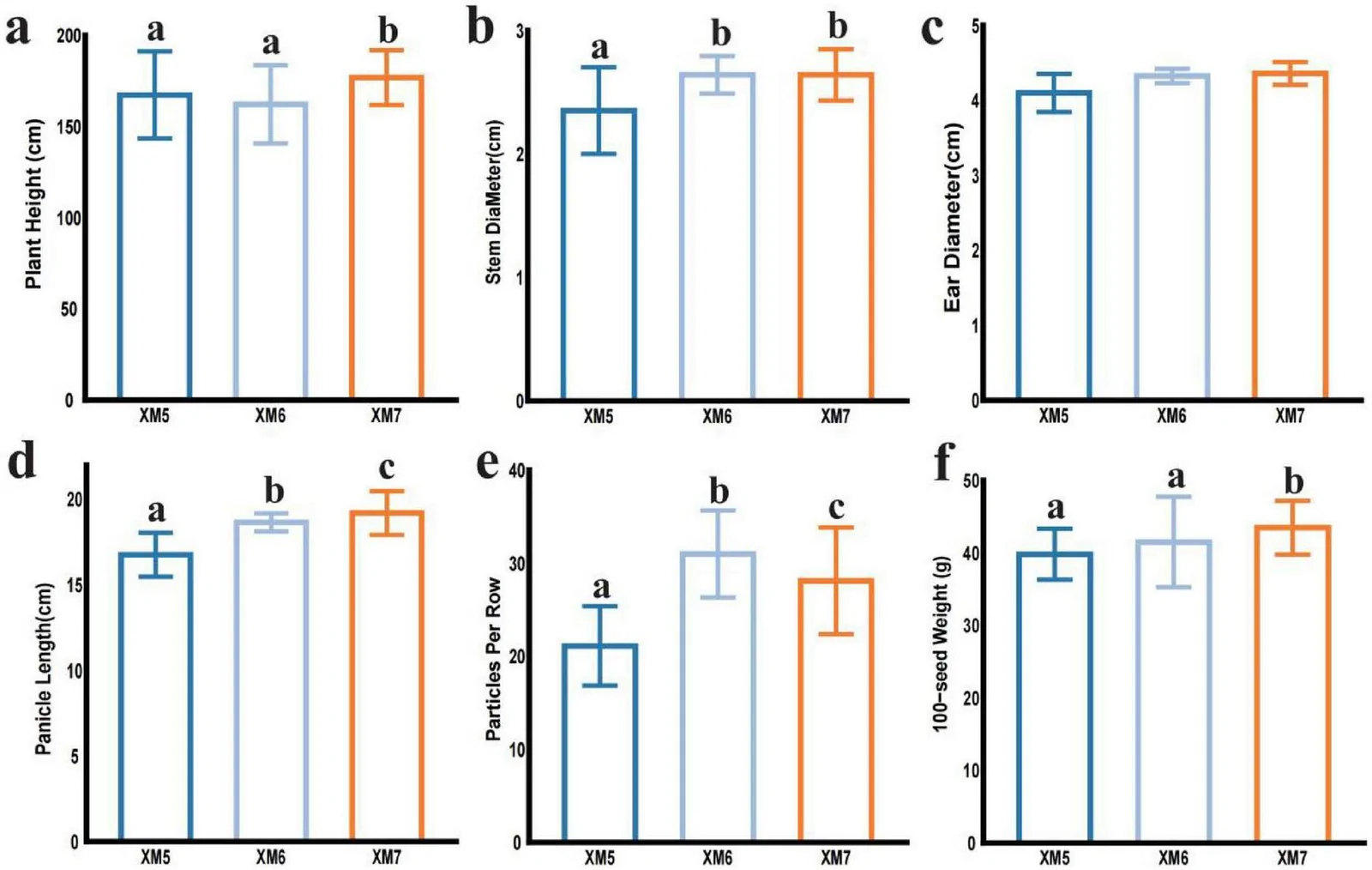
Phenotypic characterization and agronomic performance of maize in a soil acidification gradient from pot experiment, including plant height **(a)**, stem diameter **(b)**, ear diameter **(c)**, panicle length **(d)**, particles per row **(e)** and 100 seed weight **(f)**. Different letters indicate significantly different groups (*P* < 0.05, ANOVA, Tukey HSD).

Our observations revealed that maize growth is severely impeded under highly acidic conditions (pH 2.0–4.0), where soil nitrite (NO_2_^–^) concentrations were quantified at 27.78, 28.52, 17.99, 13.21, 6.52, 1.75 mg/kg at pH levels of 2.0, 3.0, 4.0, 5.0, 6.0, and 7.0, respectively. To conclusively determine whether this extreme nitrite accumulation, driven by the suppression of nitrogen-cycling microbiota in acidified soils, is the direct cause of maize mortality, we amended neutral soils with sodium nitrite (Corresponding to the amount of nitrite in soil after acidification) to mirror the exact concentrations observed undersoil. To disentangle whether the toxicity in highly acidic soils stems directly from chemical proton stress or from nitrogen transformation bottlenecks, we quantified soil inorganic nitrogen fractions. In acidic treatments (pH 2.0–4.0), soil nitrite (NO_2_^–^-N) concentrations dramatically accumulated to 27.78, 28.52, and 17.99 mg kg^–1^, whereas in neutral soil (pH 7.0), NO_2_^–^-N remained at a baseline level of 1.75 mg kg^–1^ (Figure 2i). To directly test the phytotoxicity of accumulated NO_2_^–^-N independent of proton stress, neutral soils (pH 7.0) were spiked with exogenous sodium nitrite (NaNO_2_) matching the observed soil concentrations (1.75–28.52 mg kg^–1^; Treatments M7–M2) (Supplementary Figure 1).

**FIGURE 2:**
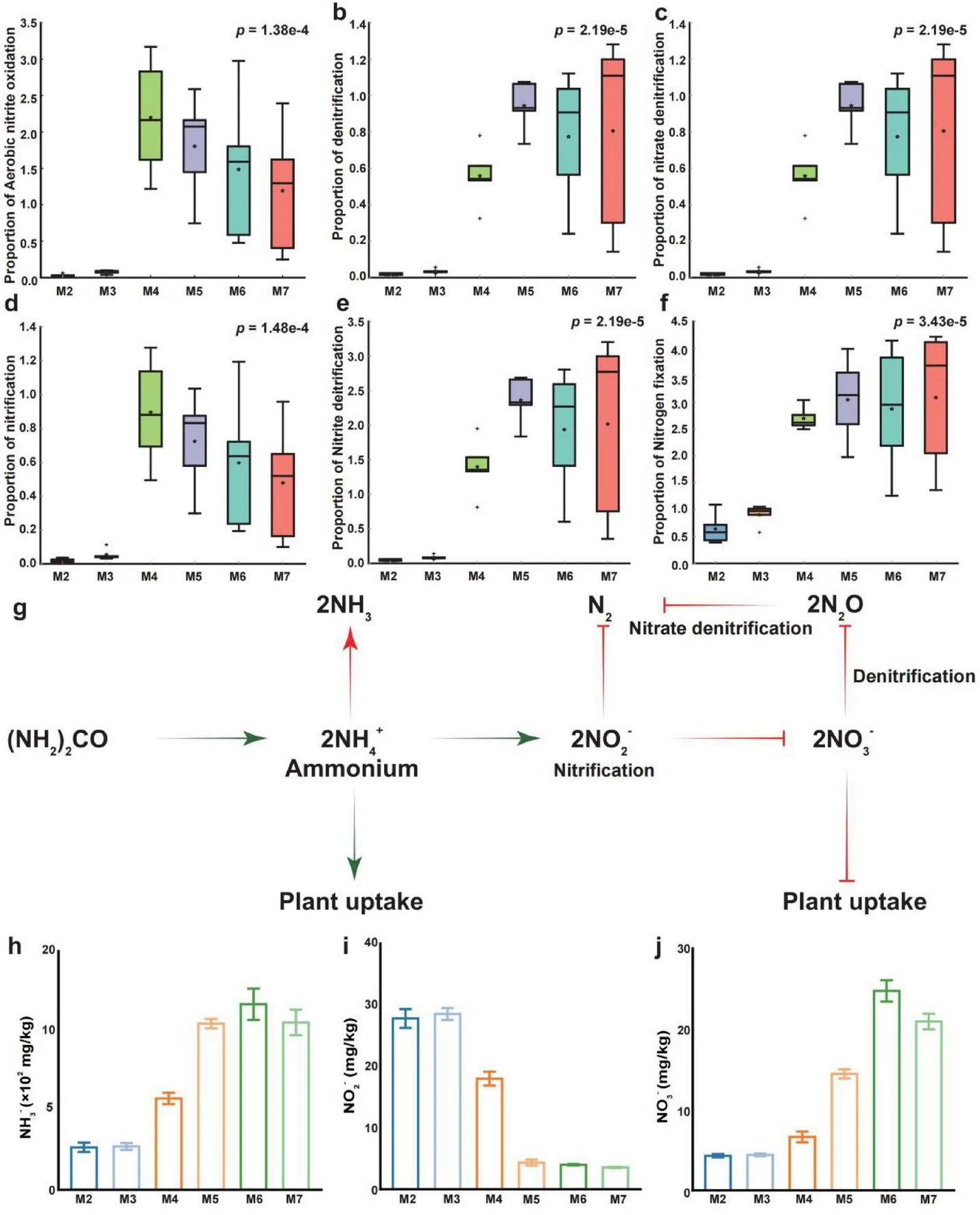
Shifts in nitrogen-transforming microbial guilds and nitrogen pools along a soil acidification gradient from pot experiment. **(a)** Abundance of aerobic nitrite oxidation. **(b)** Abundance of denitrification. **(c)** Abundance of nitrate denitrification. **(d)** Abundance of nitrification. **(e)** Abundance of nitrite denitrification. **(f)** Abundance of nitrogen fixation. **(g)** Functional architecture of nitrogen metabolism under soil acidification stress. **(h–j)** NH_4_^+^, NO_2_^–^, NO_3_^–^ concentration from different soil acidification gradient, respectively. Green arrows represent normal nitrogen transformation processes; red arrows indicate reduced transformation rates; T-bar arrows denote inhibition of the corresponding transformation processes.

The dose-response experiments revealed a sharp, concentration-dependent inhibition of early maize development. Germination in hydroponic assays maintained high levels under low-to-moderate NO_2_^–^-N concentrations (75.00% ± 10.00% in M7 [1.75 mg kg^–1^] and 76.67% ± 10.41% in M6 [6.52 mg kg^–1^]), showing no significant difference compared to M5 (13.21 mg kg^–1^, 71.67% ± 2.89%) (Supplementary Figures 1a, b). However, exceeding 13.21 mg kg^–1^ led to severe germination suppression, dropping significantly to 55.00% ± 5.00% in M4 (17.99 mg kg^–1^) and 38.33% ± 14.43% in M3 (28.52 mg kg^–1^) (*P* < 0.05). Under the highest NO_2_^–^-N level (27.78 mg kg^–1^, M2), germination was completely arrested (0%). Vegetative traits in surviving seedlings exhibited parallel declines: plant height and leaf area decreased by 12.9% and 43.8% in M4 compared to control M7 (*P* < 0.05) (Supplementary Figures 1c–e). These findings establish that NO_2_^–^-N accumulation beyond the critical threshold of ∼13.21 mg kg^–1^ exerts severe, independent phytotoxicity on maize growth.

### 3.2 Response of rhizospheric microbial assembly to pH-driven deterministic pressure

To characterize the adaptive responses of rhizospheric microbial consortia, we conducted high-throughput Illumina sequencing of the 16S rRNA gene (V5–V7 regions). Our analysis revealed that microbial alpha diversity was significantly compromised under intensifying acidity, with communities at pH 2 and 3 exhibiting unique structural characteristics distinct from those at pH ≥ 4 (Figure 3a). Unconstrained principal coordinate analysis (PCoA) based on Bray-Curtis distances substantiated that soil pH accounted for 79.95% of the total community variance, forming four discrete clusters along the acidification gradient (Figure 3b). Furthermore, the beta nearest taxon index (βNTI) analysis demonstrated a transition in assembly processes: while stochastic environmental filtering influenced communities near neutrality, community assembly shifted toward intense deterministic selection as soil pH decreased (Figure 3c). These results underscore that acidified soils harbor a highly specialized microbial architecture shaped by rigid environmental filtering.

**FIGURE 3:**
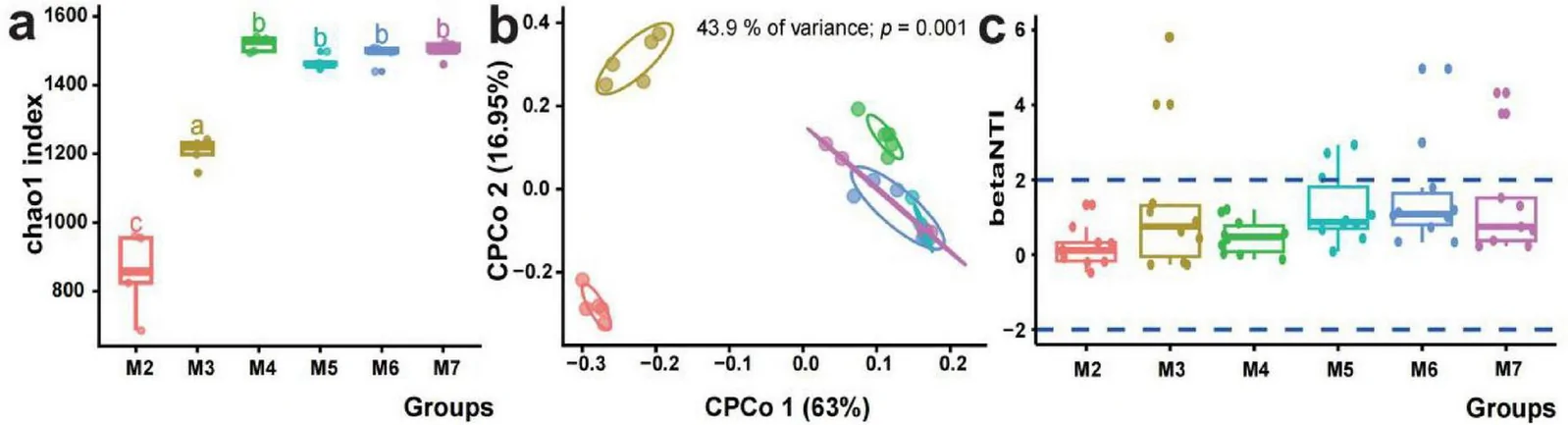
Structural responses and assembly processes of the root-associated microbiota across a soil acidification gradient (pH 2.0–7.0) from pot experiment. **(a)** Alpha diversity, represented by the Chao1 index, of root-associated microbial communities across the experimental soil acidification gradient (pH 2.0–7.0). **(b)** Unconstrained Principal Coordinates Analysis (PCoA) based on Bray–Curtis distances, illustrating the divergent clustering of root microbiota under varying acidification levels in the pot experiment. The first two axes (PCo1 and PCo2) explain the dominant variance (*P* < 0.001, PERMANOVA by Adonis). **(c)** Distribution of the weighted Beta Nearest Taxon Index (βbetaNTI) calculated for the root microbiota across the pH gradient, characterizing the shift in microbial assembly processes in response to intensified soil acidity. Different letters indicate significantly different groups (*P* < 0.05, ANOVA, Tukey HSD).

### 3.3 Shifts in nitrogen-cycling functional profiles and inorganic nitrogen pools

Functional prediction using FAPROTAX identified 35 distinct functional pathways, with nitrogen (N) cycling processes representing the dominant metabolic categories. Severe soil acidification (pH 2.0–3.0) induced a comprehensive suppression of functional guilds involved in aerobic nitrite oxidation, nitrification, denitrification, nitrate denitrification, and nitrogen fixation relative to circumneutral soils (*p* < 0.001, Figures 2a–f; *Post hoc* comparisons in Figure 4).

**FIGURE 4:**
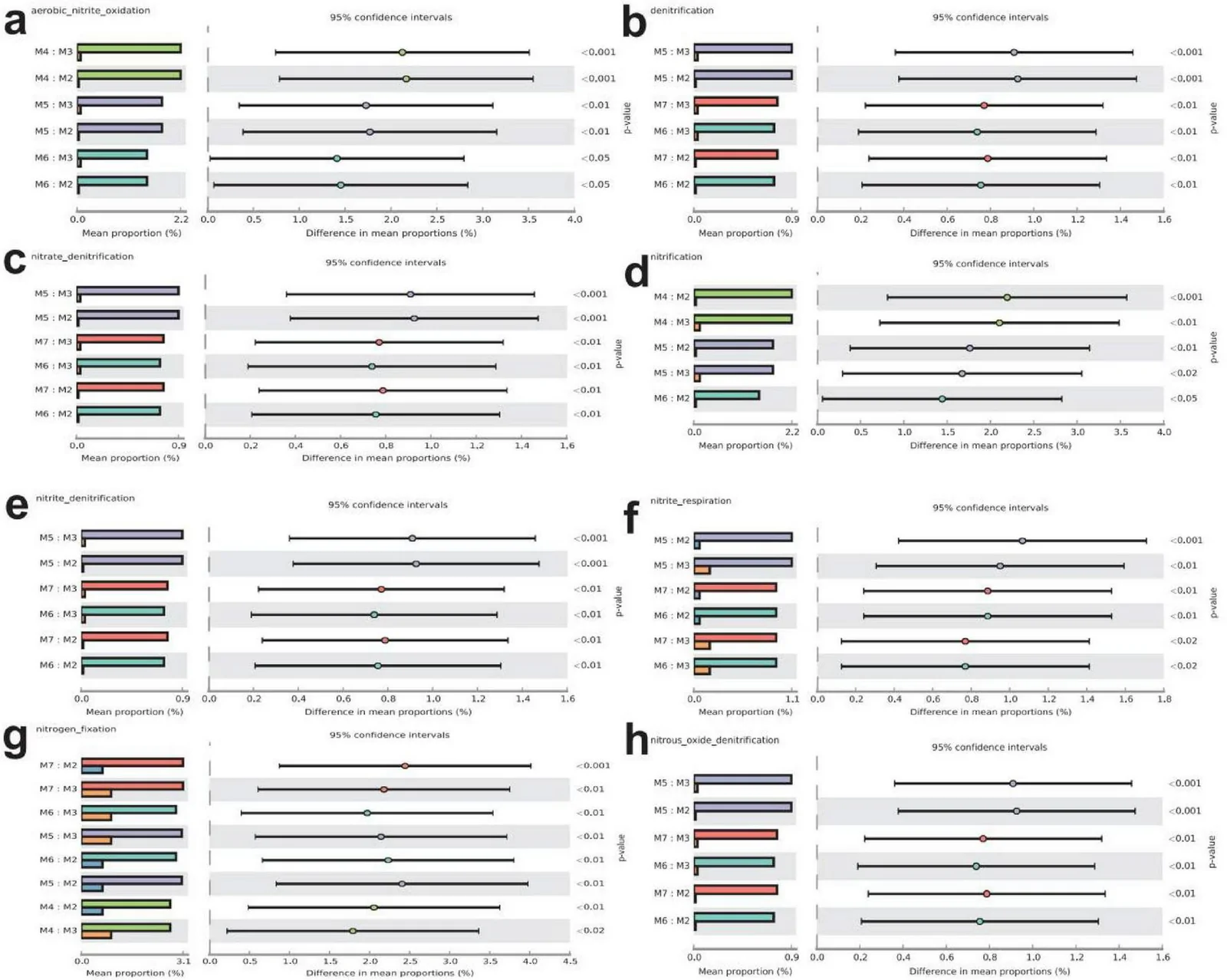
*Post hoc* comparison results of microorganisms related to soil nitrogen cycling from pot experiment. **(a)** *Post hoc* comparison results of aerobic nitrite oxidation. **(b)** *Post hoc* comparison results of denitrification. **(c)** *Post hoc* comparison results of nitrate denitrification. **(d)** *Post hoc* comparison results of nitrification. **(e)** *Post hoc* comparison results of nitrite denitrification. **(f)** *Post hoc* comparison results of nitrite respiration. **(g)** *Post hoc* comparison results of nitrogen fixation. **(h)** *Post hoc* comparison results of nitrous oxide denitrification.

Reconstruction of the N flux architecture revealed a major functional breakdown (Figure 2g). While urea hydrolysis and ammonium (NH_4_^+^) oxidation proceeded under acidic conditions, the subsequent oxidation of nitrite (NO_2_^–^) to nitrate (NO_3_^–^) was severely blocked. Furthermore, gaseous N loss pathways (denitrification and nitrous oxide denitrification) were significantly inhibited, trapping nitrogenous intermediates within the edaphic matrix. Quantification of inorganic N pools confirmed this metabolic blockage: NH_4_^+^-N and NO_3_^–^-N concentrations dropped markedly in extreme acidic soils (pH 2.0–3.0) to ∼250 mg kg^–1^ and ∼4.5 mg kg^–1^, respectively, compared to peak values of 1,402.03 mg kg^–1^ and 24.53 mg kg^–1^ in moderately acidic to neutral soils (Figure 2h, j). Crucially, NO_2_^–^-N spiked sharply to 27.78–28.52 mg kg^–1^ at pH 2.0–3.0 (Figure 2i), validating that severe acidification creates a functional bottleneck that traps nitrogen as toxic nitrite.

### 3.4 Molecular evidence for the decoupling of ammonia and nitrite oxidation

To unravel the molecular mechanism underpinning nitrite accumulation, we quantified key functional genes involved in nitrification using FAPROTAX and qPCR (Figure 5, Supplementary Figure 2). The abundance of the *amoA* gene (encoding ammonia monooxygenase subunit A) in ammonia-oxidizing archaea (*AOA*) and bacteria (*AOB*) was significantly upregulated under strong acidity (pH 2.0–4.0) compared to circumneutral soils (pH 5.0–7.0) (*p* < 0.001, Figure 5a, Supplementary Figure 2a). Similarly, amoB expression exhibited a consistent elevated trend under high acidity (Figure 5b, Supplementary Figure 2b).

**FIGURE 5:**
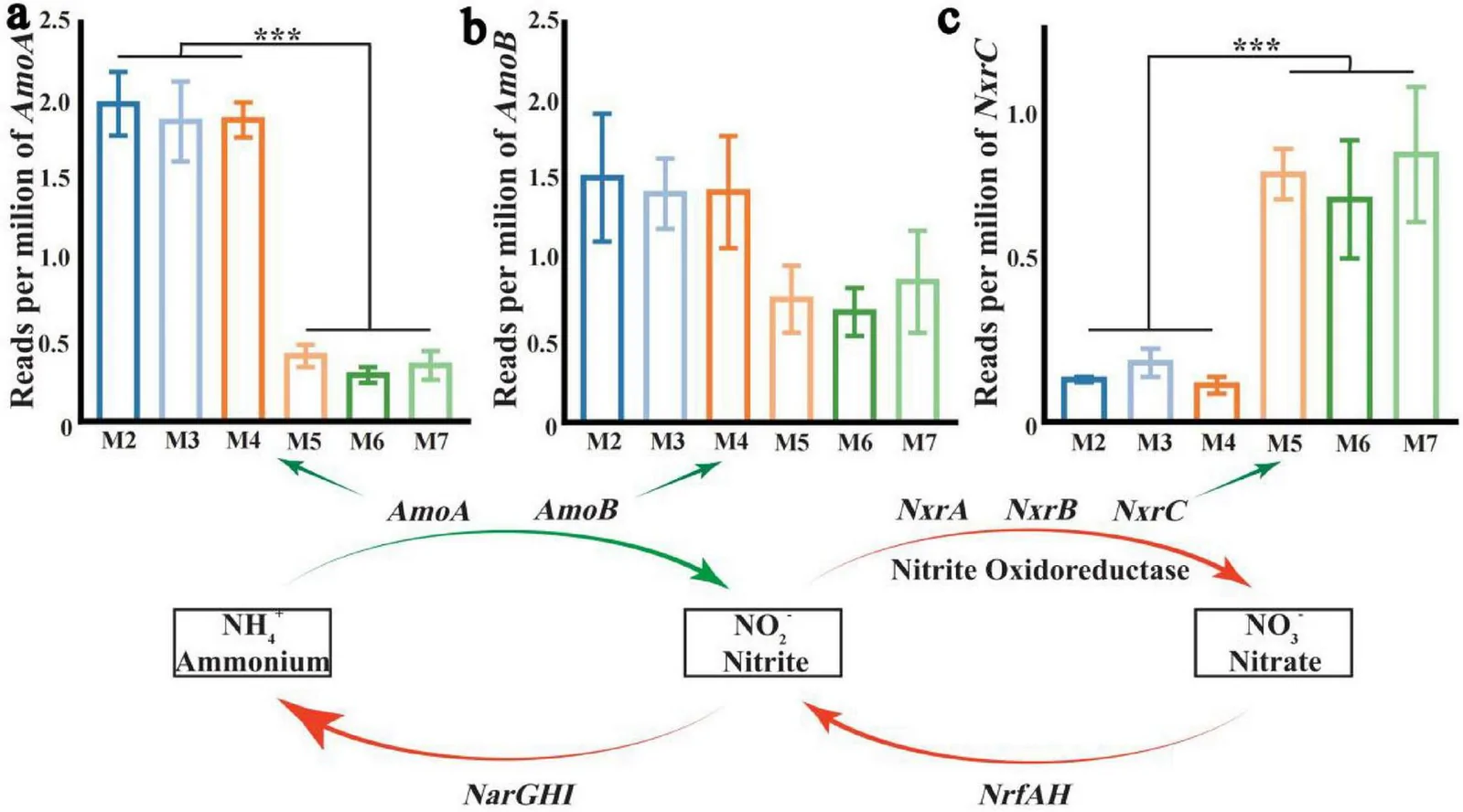
Responses of key nitrification-related genes in the root-associated microbiota to soil acidification from pot experiment. **(a)** Relative abundances of *AmoA*. **(b)** Relative abundances of *AmoB*. **(c)** Relative abundances of *NxrC*. The diagram represents microbial pathways related to the soil nitrogen cycle. The relative abundances of *AmoA* (FDR adjusted *P* = 0.031, Welch’s *t*-test) and *NxrC* (FDR adjusted *P* = 0.027, Welch’s *t*-test) were significantly less abundant in the root microbiome under low pH conditions (pH 2.0–4.0). Although the change of *AmoB* did not pass the statistical threshold after multiple test correction (FDR adjusted *P* = 0.057, Welch’s *t*-test), it showed a clear trend of increase in the acidic soil. *p < 0.05, **p < 0.001, ***p < 0.0001.

In stark contrast, the abundance and transcript level of NxrC (encoding nitrite oxidoreductase subunit C), responsible for converting NO_2_^–^ to NO_3_^–^, was severely suppressed in soils at pH 2.0–4.0 relative to pH 5.0–7.0 (*p* < 0.001, Figure 5c, Supplementary Figure 2c). This asymmetric response demonstrates a profound molecular decoupling between ammonia oxidation (*amoA*/*amoB*) and nitrite oxidation (*NxrC*). Acidification accelerates the primary step of ammonia conversion into nitrite while disabling the secondary step of nitrite neutralization, providing a direct molecular mechanism for the massive accumulation of toxic NO_2_^–^ in acidified soils.

## 4 Discussion

### 4.1 Soil acidification imposes deterministic environmental filtering on rhizosphere microbiota

Soil pH is widely recognized as a master variable shaping microbial community assembly and ecological fitness in terrestrial ecosystems (Wang and Kuzyakov, 2024a,b; Zhou et al., 2026). We propose that the profound divergence in microbial community structure is not merely a descriptive shift but a fundamental reorganization of the rhizospheric metabolic landscape, where the extreme environmental filtering observed at low pH imposes a rigid bottleneck on N turnover. This is substantiated by the βNTI analysis, which shows that as soil pH deviates from neutrality, the community assembly shifts from stochastic processes toward intense deterministic selection (Tripathi et al., 2018). Under these high-stress conditions, the survival of a few acid-tolerant taxa comes at the cost of losing critical functional guilds, particularly those responsible for nitrite oxidation and denitrification.

The convergence of our taxonomic, functional, and geochemical datasets provides a high-resolution map of how soil acidification reconfigures the rhizospheric interactome, moving beyond simple diversity loss to a state of functional truncation. We demonstrate that the deterministic environmental filtering quantified by βNTI values approaching +2 in extremely acidic soils aligns precisely with a catastrophic collapse in nitrogen-transforming microbial guilds. The dramatic accumulation of nitrite (NO_2_^–^) to 20.78 mg/kg serves as a geochemical signature of the inhibited nitrite-oxidizing consortia and a direct source of phytotoxicity in maize. Consequently, restoring the ecological functionality of these acidified systems necessitates strategies aimed directly at mitigating soil nitrite accumulation and relieving metabolic bottlenecks in the N cycle.

### 4.2 Molecular decoupling of nitrifying guilds drives a functional nitrogen bottleneck

The most striking finding of this study is the massive enrichment of soil nitrite (NO_2_^–^-N, reaching ∼28.5 mg kg^–1^) in highly acidified Mollisols (Figure 2i). Through integrated functional profiling (FAPROTAX) and gene quantification (qPCR/metagenomics), we resolved the precise molecular bottleneck driving this phenomenon (Figure 5). Nitrification is traditionally viewed as a tightly coupled two-step process executed by ammonia oxidizers (*AOA/AOB*) and nitrite-oxidizing bacteria (*NOB*) (Séneca et al., 2020). However, our data demonstrate that soil acidification induces a profound physiological and transcriptional decoupling between these two functional cohorts.

Specifically, ammonia monooxygenase genes (*amoA* and *amoB*) remained active or were significantly upregulated under acidic conditions (pH 2.0–4.0) (Figures 5a, b), consistent with the higher acid tolerance reported for certain *AOA* lineages (e.g., Nitrosotalea) that thrive in low-pH edaphic environments. Conversely, the expression of *NxrC*, encoding the key subunit of nitrite oxidoreductase in *NOB*, was near-completely suppressed at pH < 4.0 (Figure 5c). *NOB* (such as *Nitrobacter* and *Nitrospira*) are notoriously sensitive to proton toxicity and nitrous acid (HNO_2_) inhibition. Concurrently, FAPROTAX analysis indicated that gaseous nitrogen loss pathways (denitrification) were simultaneously suppressed (Figures 2b–e). Thus, acid-induced inhibition of *NOB* prevents NO_2_^–^ conversion to NO_3_^–^, while denitrification failure prevents NO_2_^–^ dissipation as gaseous N_2_, trapping nitrogen in the form of NO_2_^–^ within the soil solution.

### 4.3 Nitrite accumulation as an overlooked phytotoxic driver of maize failure

Agronomic yield loss in acidic soils has historically been attributed primarily to toxic trivalent aluminum ions (Al^3+^) or manganese (Mn^2+^) toxicity, alongside phosphorus deficiency. While Al^3+^ toxicity undoubtedly impairs root cell elongation and disrupt root morphological development, our exogenous NaNO_2_ spiking experiments in neutral soil (pH 7.0) effectively eliminated the interference of H^+^, Al^3+^, and Mn^2+^ stress, under this controlled setting, definitively verifying that soil NO_2_^–^ accumulation acts as an independent, high-efficiency phytotoxic pathway that severely suppresses maize growth (Supplementary Figure 1). It should be emphasized that this neutral-soil spiking assay only mimics stress from ionic NO_2_^–^. In real-world field acidified soils, plants face combined co-occurring stressors including H^+^, Al^3+^, HNO_2_, and NO_2_^–^, whose relative contributions to crop damage cannot be quantitatively disentangled based on our present dataset. Exogenous NO_2_^–^ exposure above 13.21 mg kg^–1^ triggered severe physiological collapse, causing dramatic declines in germination, stem diameter, and leaf expansion, with complete seedling death occurring at 27.78 mg kg^–1^ NO_2_^–^ (matching the concentration in pH 2.0 soil).

Nitrite toxicity in plants operates via multiple damaging mechanisms. Upon root uptake, NO_2_^–^ is protonated to form nitrous acid (HNO_2_, pKa = 3.25) in acidic apoplastic environments, which readily diffuses across cell membranes, causing intracellular acidification, lipid peroxidation, and destruction of photosynthetic pigments Furthermore, excess intracellular NO_2_^–^ inhibits root cytochrome coxidase, severely impairing mitochondrial respiration and active nutrient transport. Our findings demonstrate that microbially driven NO_2_^–^ toxification is a primary mechanism underlying crop death in acidified agricultural lands.

In contrast, the charged NO_2_^–^ ion cannot freely penetrate complete cell membranes and is primarily retained in the root apoplast and extracellular space after root uptake. A portion of residual NO_2_^–^ eventually enters the cell cytoplasm via root ion transport channels, exerting unique metabolic toxicity independent of HNO_2_ stress. Intracellular excess NO_2_^–^ specifically inhibits the activity of cytochrome coxidase, a key rate-limiting enzyme in the mitochondrial aerobic respiratory chain of maize roots. This inhibitory effect disrupts mitochondrial energy metabolism, severely impairs root respiratory function, and further blocks the active absorption and directional transport of mineral nutrients, ultimately resulting in stunted maize seedling growth.

### 4.4 Ecological management and mitigation strategies for acidified agroecosystems

Discovering the nitrite-mediated bottleneck provides actionable insights for managing acidic soils and restoring nitrogen use efficiency. First, conventional fertilization strategies that heavily rely on high doses of synthetic ammonium-based fertilizers or urea accelerate proton release via the initial amoA-driven step, exacerbating NO_2_^–^ accumulation. To break this toxic feedback loop, nitrogen application rates must be dynamically optimized and synchronized with crop demand.

Second, soil remediation must shift from sole chemical neutralization (e.g., lime application) to holistic microbiome restoration. Incorporating biochar or organic amendments with high cation exchange capacity can raise soil pH while providing micro-habitats that support the recovery of acid-sensitive *NOB* communities. Bio-augmentation with specialized, acid-tolerant nitrite-oxidizing strains or biostimulants represents another promising avenue to relieve the *NxrC* transcription bottleneck, preventing toxic NO_2_^–^ build-up and restoring sustainable crop productivity in vulnerable Mollisol regions.

## 5 Conclusion

In conclusion, this study identifies a microbially mediated nitrite toxification bottleneck as a key driver of maize failure in acidified soils. Acidification imposes strong deterministic selection on the rhizosphere microbiome, causing a molecular decoupling between ammonia oxidation (*amoA*) and nitrite oxidation (*NxrC*). The resulting hyper-accumulation of NO_2_^–^ (>13.21 mg kg^–1^) acts as an independent phytotoxin, severely impairing maize growth and germination. Restoring soil health in acidified agroecosystems requires integrated strategies combining balanced N fertilization, soil buffering amendment, and functional restoration of nitrite-oxidizing microbial guilds.

## Data Availability

The datasets presented in this study can be found in online repositories. The names of the repository/repositories and accession number(s) can be found in this article/Supplementary material.
