# Supplementary material for: Soil acidification drives a massive accumulation of nitrite as a mechanistic bottleneck to inhibit maize growth and productivity
Source: Front Microbiol. 2026 Sep 18;17:1830432. doi: 10.3389/fmicb.2026.1830432 (PMC13630935; doi:10.3389/fmicb.2026.1830432)
Supplement: Supplementary file 1 [file Supplementary_file_1.pdf]

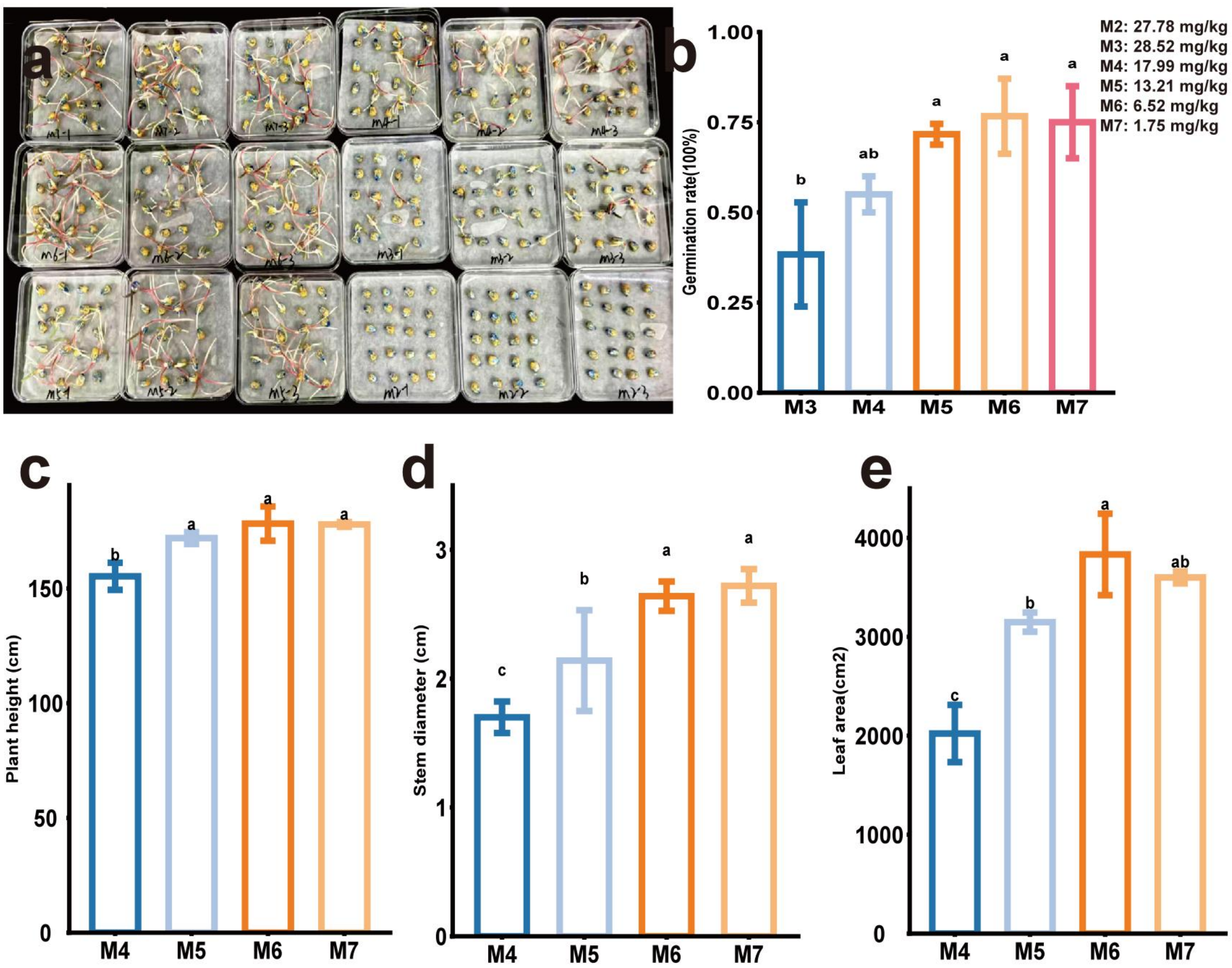


**Fig. S1 | In order to understand the toxicity of $NO_2^-$ at different doses ( 27.78, 28.52, 17.99, 13.21, 6.52, 1.75 mg/kg at pH levels of 2.0, 3.0, 4.0, 5.0, 6.0, and 7.0), the $NO_2^-$ at different doses were added to soil from hydroponic germination.** (a) and (b) Germination rate in hydroponics, (c) Plant height (cm), (d) Stem diameter, and (e) Leaf area. The different letters above the columns indicate a significant difference ($P < 0.05$) based on the analysis of variance. Different letters indicate significantly different groups ($P < 0.05$, ANOVA, Tukey HSD).

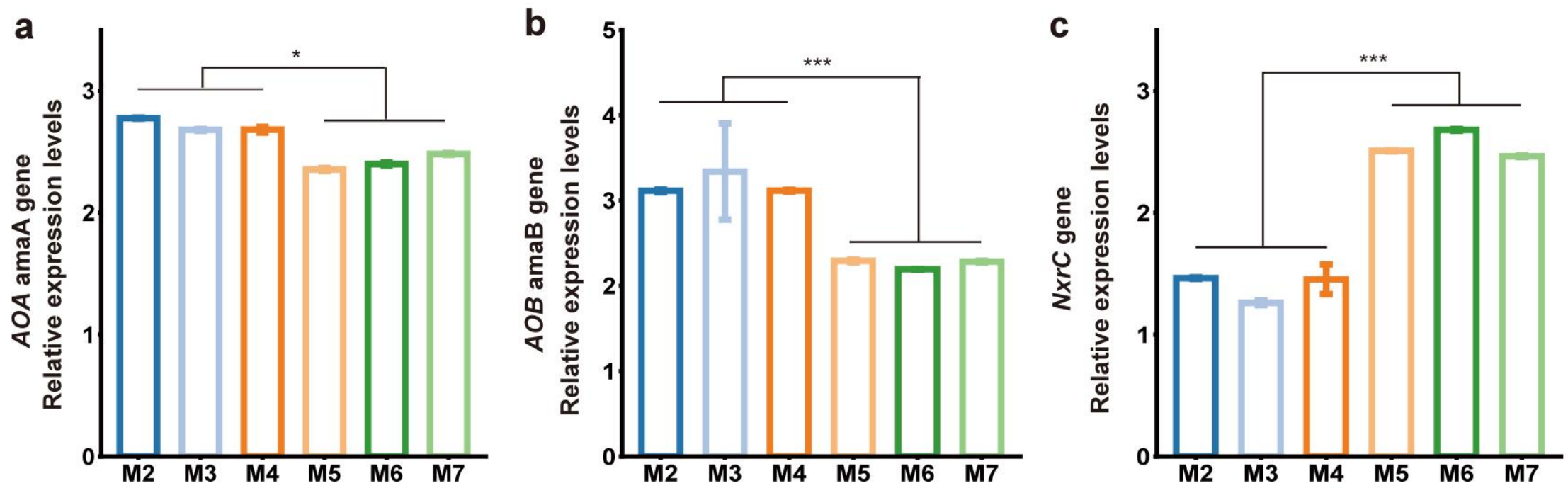


**Fig. S2 | Quantitative RT-PCR analysis of related gene including** (a) *AOA*, (b) *AOB* and (c) *NxrC* from pot experiment. Error bars represent the standard errors of the triplicate microcosms. The different letters above the columns indicate a significant difference ($P < 0.05$) based on the analysis of variance.
