# Supplementary material for: Soil acidification drives a massive accumulation of nitrite as a mechanistic bottleneck to inhibit maize growth and productivity
Source: Front Microbiol. 2026 Sep 18;17:1830432. doi: 10.3389/fmicb.2026.1830432 (PMC13630935; doi:10.3389/fmicb.2026.1830432)
Supplement: Supplementary file 2 [file Table_1.docx]

**Analysis of Soil Physicochemical Properties**

Characterization of Soil Physicochemical Properties and Experimental DesignThe experimental substrate was a typical Mollisol (Black soil) collected from Northeast China, a region critical for maize production. To provide a comprehensive baseline of the edaphic environment, key physicochemical properties were systematically characterized and are summarized in Supplementary Table S1. Specifically, soil pH was determined using a glass electrode at a 1:2.5 soil-to-water ratio (w/v); total nitrogen (TN) and soil organic matter (SOM) were quantified via the dry combustion method (elemental analyzer) and the K_2_Cr_2_O_7_ oxidation method, respectively. Cation exchange capacity (CEC) was measured using the ammonium acetate exchange method, while available phosphorus (AP) and available potassium (AK) were extracted by the Olsen and flame photometry methods.To simulate a precise acidification gradient, we established a series of pH-regulated microcosms. Deionized water was meticulously titrated with glacial acetic acid to generate irrigation solutions with pH values ranging from 2.0 to 7.0. Each pot, containing a uniform mass of the aforementioned Mollisol, was saturated with 2,000 mL of the respective pH-adjusted solution. To ensure the edaphic acidity reached a stable equilibrium and precisely matched the target experimental design, soil pH dynamics were monitored in real-time using in situ pH sensors (e.g., IQ Scientific Instruments) until a stabilized pH range of 2.0 to 7.0 was consistently achieved across treatments.The biological component utilized maize seeds provided by Shenyang Agricultural University. Following the stabilization of the soil chemical environment, seeds were sown at a density of three per hole in the pre-conditioned microcosms. To mitigate stochastic environmental effects and ensure high statistical power for downstream microbial and phenotypic analyses, each pH treatment was conducted with five independent biological replicates (n=5). This rigorous experimental framework allows for a robust assessment of the deterministic effects of soil acidification on the tripartite interactions between soil chemistry, microbial community assembly, and plant health.
