# Supplementary material for: Soil acidification drives a massive accumulation of nitrite as a mechanistic bottleneck to inhibit maize growth and productivity
Source: Front Microbiol. 2026 Sep 18;17:1830432. doi: 10.3389/fmicb.2026.1830432 (PMC13630935; doi:10.3389/fmicb.2026.1830432)
Supplement: Supplementary file 3 [file Table_2.docx]

**Table S1. Physical and chemical properties of soil**

| Soil | PH | CE（μs/cm) | TOC(mg/L) | IC(mg/L) | TC(mg/L) | TN(mg/L) | N(%) | C(%) |
| --- | --- | --- | --- | --- | --- | --- | --- | --- |
| M7 | 6.59 | 1959.67 | 50.67 | 0.80 | 322.00 | 1.27ab | 0.27a | 1.95b |
| M6 | 6.08 | 2970.67 | 62.43 | 0.58 | 306.59 | 1.20ab | 0.26a | 1.72d |
| M5 | 4.97 | 1908.00 | 48.22 | 2.92 | 215.79 | 1.12b | 0.26a | 1.81cd |
| M4 | 3.69 | 1440.67 | 45.29 | 1.35 | 169.72 | 1.30ab | 0.26a | 1.90bc |
| M3 | 2.73 | 1673.00 | 46.68 | 0.54 | 128.97 | 1.40a | 0.27a | 2.11a |
| M2 | 2.03 | 1343.00 | 45.38 | 0.54 | 142.11 | 1.40a | 0.27a | 2.11a |

Note: CE stands for conductivity; TOC stands for total organic carbon;IC stands for inorganic carbon;TC stands for total carbon;TN stands for total nitrogen.

**Table S2. Elements concentration of soil**

| **Treatment** | **Na (g/kg)** | **Mg (g/kg)** | **P (g/kg)** | **K (g/kg)** | **Ca (g/kg)** | **Fe (g/kg)** | **B (mg/kg)** | **S (mg/kg)** | **Mn (mg/kg)** | **Cu (mg/kg)** | **Zn (mg/kg)** | **Mo (mg/kg)** |
| --- | --- | --- | --- | --- | --- | --- | --- | --- | --- | --- | --- | --- |
| M7 | 0.51 | 3.88 | 1.35 | 11.16 | 1.79 | 64.49 | 110.23 | 397.69 | 796.98 | 41.94 | 51.72 | 0.93 |
| M6 | 0.54 | 3.62 | 1.94 | 10.69 | 1.70 | 57.96 | 108.54 | 371.79 | 837.68 | 42.96 | 46.13 | 1.19 |
| M5 | 0.50 | 3.72 | 1.29 | 11.67 | 1.88 | 53.73 | 126.84 | 321.24 | 808.84 | 41.15 | 52.15 | 1.38 |
| M4 | 0.43 | 3.34 | 1.59 | 12.52 | 1.57 | 54.56 | 117.46 | 359.53 | 940.58 | 42.37 | 49.28 | 1.25 |
| M3 | 0.35 | 3.59 | 2.09 | 10.83 | 1.35 | 42.44 | 125.57 | 360.93 | 827.82 | 34.75 | 40.12 | 1.31 |
| M2 | 0.37 | 3.59 | 2.09 | 10.83 | 1.35 | 45.32 | 124.97 | 275.25 | 757.42 | 31.35 | 39.30 | 1.21 |

**Table S3. Seedling Germination and Acute Mortality Rates Across the Nitrite Validation Gradient (pH 7.0)**

| NaNO_2_  (mg kg^-1^) | Treatment Description | Germination Rate (%) | Acute Mortality Rate (after 2 hours) (%) | Phenotypic Observation / Crop Outcome |
| --- | --- | --- | --- | --- |
| 0 | pH 7 Soil + Water Only (Control) | 100.0 ± 0.0 | 0.0 ± 0.0 | Healthy growth; vigorous root elongation; no necrosis |
| 5 | pH 7 Soil + Low Only NO_2_^-^ (Control) | 100.0 ± 0.0 | 0.0 ± 0.0 | Slight reduction in root elongation rate, no death |
| 10 | Neutral Soil + Medium NO_2_^-^ | 93.3.0 ± 4.5 | 6.7 ± 5.8 | Leaf tip curling; partial chlorosis |
| 20 | Neutral Soil + High NO_2_^-^ | 0.0 ± 0.0 | 100.0 ± 0.0 | Total seedling mortality; systemic root necrosis within 2 h |
